# The Basis of Cognitive and Behavioral Dysfunction in Amyotrophic Lateral Sclerosis

**DOI:** 10.1002/brb3.70115

**Published:** 2024-11-05

**Authors:** Alexander Bampton, Caroline McHutchison, Kevin Talbot, Michael Benatar, Alexander G. Thompson, Martin R. Turner

**Affiliations:** ^1^ Nuffield Department of Clinical Neurosciences University of Oxford Oxford UK; ^2^ Division of Psychology University of Stirling Stirling UK; ^3^ Department of Neurology University of Miami Miller School of Medicine Miami Florida USA

**Keywords:** amyotrophic lateral sclerosis, behavioral, C9orf72, cognitive, frontotemporal dementia, motor neurone disease, TDP‐43

## Abstract

**Objective:**

To summarize and evaluate evidence pertaining to the clinical, genetic, histopathological, and neuroimaging correlates of cognitive and behavioral dysfunction in amyotrophic lateral sclerosis (ALS).

**Methodology:**

We comprehensively reviewed the literature on cognitive and behavioral manifestations of ALS, narrating findings from both cross‐sectional and longitudinal studies. We discussed knowledge gaps in the evidence base and key limitations affecting studies to date, before formulating a framework for future research paradigms aimed at investigating clinicopathological correlates of neuropsychological dysfunction in ALS.

**Results:**

Studies have demonstrated clinical associations with cognitive dysfunction in ALS e.g., bulbar‐onset of symptoms, pathological associations (extramotor TDP‐43 deposition), and imaging associations (frontotemporal involvement). The most common behavioral deficit, apathy, is highly associated with verbal fluency, but longitudinal studies assessing behavioral dysfunction in ALS are comparatively lacking.

**Conclusion:**

Longitudinal studies have been helpful in identifying several potential correlates of cognitive and behavioral dysfunction but have frequently been confounded by selection bias and inappropriate testing platforms. This review provides a framework for more robust assessment of clinicopathological associations of neuropsychological abnormalities in ALS in the future, advocating for greater utilization of pre‐symptomatic *C9orf72* repeat expansion‐carrying cohorts.

## Graphical abstract

This review examines the clinical, pathological, and imaging associations of cognitive and behavioral dysfunction in ALS, with a critique of recent longitudinal studies. It advocates for more robust protocols for assessing clinicopathological correlates of neuropsychological abnormalities in the future, particularly using pre‐symptomatic *C9orf72* repeat expansion‐carrying cohorts.

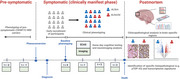

## 1 Introduction

Amyotrophic lateral sclerosis (ALS) was characterized by Jean‐Martin Charcot (1825–1893) as a progressive syndrome involving the combined degeneration of upper motor neurons of the corticospinal tract and lower motor neurons of the brain stem and anterior spinal cord. The resulting progressive skeletal muscle weakness strongly underpins the median survival of 30 months from the onset of symptoms (Westeneng et al. 2018). The categorization of ALS as solely a disorder of voluntary movement has been revealed as an oversimplification. ALS has clinical, pathological, and genetic overlap with frontotemporal dementia (FTD), with a shared signature of neuronal and glial cytoplasmic inclusions of TDP‐43 (Neumann et al. 2006). The emergence of an anterior brain component to ALS pathology goes back more than a century (reviewed in Turner and Swash 2015). Early neuropsychological studies reporting cognitive impairments in ALS (Gallassi et al. 1985) have developed over the last four decades into a more complex picture encompassing both cognitive and behavioral components within a multisystem syndrome (Abrahams 2023). The precise distinction of the terms cognitive and behavioral is debatable, but in recognizing the broader phenotypic spectrum, consensus criteria involving both terms have been established for the conceptualization and diagnosis of frontotemporal dysfunction in ALS (Strong et al. 2017; Table 1).

**TABLE 1:** Diagnostic classification and characteristics of amyotrophic lateral sclerosis (ALS)–frontotemporal spectrum disorder (FTSD) subtypes (Strong et al. 2017).

Acronym	ALS–FTSD phenotype	Definition/Characteristics
ALS	Cognitively and behaviorally normal (“pure”) ALS	Classical ALS is defined as a progressive motor system disorder with both upper and lower motor neuron involvement with an absence of cognitive or behavioral dysfunction *Not strictly a neuropsychological subtype according to Strong* et al. *criteria, but often denoted ALScn (cognitively normal) and/or ALSbn (behaviorally normal) in wider literature*
ALSci	ALS with cognitive impairment	Diagnostic criteria for ALS and evidence of concomitant executive dysfunction and/or language dysfunction
ALSbi	ALS with behavioral impairment	Diagnostic criteria for ALS and evidence of concomitant apathy or two non‐overlapping features from the Rascovsky criteria (Rascovsky et al. 2011) for behavioral variant frontotemporal dementia (FTD) (bvFTD), including disinhibition, compulsive behaviors, hyperorality, and loss of empathy
ALScbi	ALS with combined cognitive and behavioral impairment	Fulfillment of diagnostic criteria for both ALSci and ALSbi as above
ALS‐FTD	ALS with FTD	Fulfillment of diagnostic criteria for both ALS and FTD (bvFTD and/or language impairment phenotypes) or at least two features from the Rascovsky criteria (Rascovsky et al. 2011) with loss of insight and/or symptoms of psychosis
ALS‐D	ALS with dementia (not typical of FTD)	ALS in association with Alzheimer's disease, vascular dementia, or other mixed‐dementia not consistent with a diagnosis of FTD
FTD–MND‐like	FTD with evidence of motor neuron degeneration	A neuropathological diagnosis of primarily FTLD with evidence of motor neuron degeneration (MND), insufficient for a full ALS diagnosis

Estimates of the prevalence of cognitive dysfunction in ALS have varied considerably from 16% to 65% (Rusina, Vandenberghe, and Bruffaerts 2021; McMillan et al. 2022). The extent of cognitive dysfunction is also highly diverse, spanning a spectrum from mild cognitive impairment to overt FTD. The proposed continuity of this spectrum remains the subject of debate (Rusina, Vandenberghe, and Bruffaerts 2021). A proportion (10%) of those diagnosed with ALS according to motor features will have concurrent FTD with a strikingly similar neuropsychological profile to that of behavioral‐variant FTD (Strong et al. 2009). However, frontotemporal deficits in executive functioning and language are also common in ALS with only mild cognitive dysfunction (Goldstein and Abrahams 2013; Rusina, Vandenberghe, and Bruffaerts 2021). The most consistent cognitive faculty to deteriorate in ALS is verbal fluency, and more specifically, letter fluency, also known as phonemic fluency. Often measured by the ALS‐adapted verbal fluency index, letter fluency deficit is characterized by protracted ‘thinking time’ in order to generate words that begin with a specific letter (Abrahams et al. 2000; Abrahams 2023). Deficits in executive functioning (e.g., working memory, attention, and social cognition) and language (e.g., spelling and naming) are also common cognitive manifestations in ALS (Abrahams 2013; Kasper et al. 2015; Bora 2017; Ceslis et al. 2020; Pinto‐Grau et al. 2021; Palumbo et al. 2022). Notably, letter fluency relies on several executive function processes, and although it is a sensitive test of cognitive dysfunction in ALS disease, the precise reason for this remains elusive (Abrahams 2023).

Behavioral abnormalities in ALS may manifest either in isolation or alongside cognitive impairment, classified as ALSbi and ALScbi, respectively. In an analogous fashion to cognitive dysfunction, ALSbi may show behavioral abnormalities resembling the behavioral variant FTD neuropsychological profile, including apathy or at least two symptoms of disinhibition, perseverative behavior, loss of empathy, and hyperorality. Of these, apathy is by far the most commonly reported symptom in ALS affecting up to a third of individuals, as either self‐reported or by a close friend, relative, or caregiver (Kutlubaev et al. 2023). Apathy is itself a multidimensional syndrome with three major subtypes: initiation, emotional, and executive apathy, defined, respectively, as a lack of motivation for self‐generation of thoughts, emotional, and goal‐directed behaviors (Radakovic et al. 2016). Initiation apathy has emerged as the dominant subtype associated with ALS, although emotional blunting and problems in goal management are not uncommon behavioral manifestations of the disease (Radakovic et al. 2020; Caga et al. 2021).

The ability to identify those with ALS at risk of developing cognitive and behavioral impairment and to better predict their disease course would hold significant medical and psychosocial benefits. Cognitive dysfunction is associated with both reduced patient quality of life and additional caregiver burden (Burke et al. 2015; Bock et al. 2017). Behavioral change and in particular apathy and disinhibition have also been identified as predictors of high caregiver burden, even more so than physical disability (Lillo, Mioshi, and Hodges 2012; Burke et al. 2015; Caga et al. 2019). Hence, being able to better predict ALS‐related neuropsychological dysfunction could inform advanced decision making and care planning directives. Mechanistically, clinicopathological research studies may also unveil novel pathways for ALS‐specific decline as well as therapeutic candidates with important ramifications for clinical trial enrollment.

## 2 Cognitive Dysfunction

The relationship between cognitive dysfunction and ALS raises important questions with implications for care. Are particular ALS clinical phenotypes more or less associated with cognitive impairment? Does cognitive status remain stable after diagnosis, or do individuals experience decline, and how might this affect their survival? Current evidence addressing these questions arises predominantly from cross‐sectional studies together with some longitudinal cognitive assessment study paradigms.

### 2.1 Bulbar Dysfunction

Cognitive impairment is more frequent and more severe in ALS with bulbar involvement (Abrahams et al. 1997; Strong et al. 1999; Trojsi et al. 2016; Crockford et al. 2018a). Studies focusing on bulbar function as a site of first motor symptom onset (rather than bulbar involvement at any phase of disease) have produced mixed findings. This may be, in part, due to differences in the time span between disease onset and cognitive assessment, and the fact that many with limb‐onset of motor symptoms go on to develop bulbar symptoms as the disease progresses. A meta‐analysis by Yang et al. (2021) found a significant overall association between bulbar onset and cognitive impairment. However, the included studies exhibit markedly different definitions of cognitive impairment and use diverse test batteries, raising concerns about the validity of combining the results in a meaningful way. Additionally, several studies did not demonstrate adequate control for dysarthria, a key confounding factor discussed in the next section.

Several explanations have been proposed, with one possibility that bulbar involvement simply reflects an anatomically more advanced disease state (Crockford et al. 2018a). Alternate theories propose a more direct neuropathological basis for the association, suggesting a neuroanatomical overlap between affected cortical areas governing cognition (prefrontal cortex) and those regulating bulbar functions (facial, speech, and swallowing muscle control) (Brettschneider et al. 2013; Schmidt et al. 2016). Neither explanation need be mutually exclusive, but there is additional evidence for the latter in that more severe cognitive impairment has also been identified in bulbar‐onset cases (Schreiber et al., 2005) and in the presence of bulbar involvement at all disease stages (Chiò et al. 2019). Future inter‐disciplinary study designs, including imaging and, where possible, neuropathological examination, will be needed to confirm whether such structural or pathological relationships indeed exist.

### 2.2 Confounds to Cognitive Assessment

The assessment of cognitive function in ALS has been at best challenging and at worst confounded in many studies to date. Until recently, the standard battery of neuropsychological tests for those with ALS has been inadequately modified for individuals with dysarthria and limb weakness. This, coupled with a lack of standardization in test choice, definition of cognitive impairment (prior to the introduction of the ALSci/ALSbi criteria), and exclusion criteria, has likely contributed to significant lack of reproducibility seen between studies and wide range of reported frequencies of impairment. The introduction of the Edinburgh Cognitive and Behavioural ALS Screen (ECAS) as an accessible, ALS‐specific multidomain assessment was, therefore, a welcome development capable of quickly and sensitively measuring cognitive impairment in ALS (Abrahams et al. 2014; Niven et al. 2015; Strong et al. 2017). Recent studies have utilized the ECAS to verify an association between bulbar and cognitive dysfunction in ALS (Crockford et al. 2018a).

Another concern is how to reliably assess cognitive functioning over time. Practice effects are common in neuropsychological assessment, especially when follow‐up periods are short, and may lead to an underestimation of decline. Improvements in ECAS cognition scores consistent with practice effects have been shown when using a single version of the ECAS (ECAS‐A) (Crockford et al. 2018c; Poletti et al. 2018), prompting the development of alternate versions (ECAS‐B and ECAS‐C; Crockford et al. 2018b). Furthermore, reliable change indices (Crockford et al. 2018b) have been published to help identify clinically meaningful changes on an individual level. However, some improvement in scores may still be observed (Costello et al. 2021), warranting careful future consideration. Notably, the ECAS is currently available in 26 languages, but translated alternate versions, with evidence of equivalency to ECAS‐A and associated reliable change indices, are less widely available.

### 2.3 Is Cognitive Dysfunction Progressive?

Many longitudinal studies conducted over the last few decades have been dedicated to unraveling the temporal dynamics of cognitive status and decline throughout the course of ALS. However, despite numerous longitudinal studies on this issue, whether or not cognitive impairment is static or progressive remains contentious (Consonni et al. 2021; De Marchi et al. 2021). Some have concluded that impairment does not significantly worsen over time (Kasper et al. 2015; Bock et al. 2017; Poletti et al. 2018; Trojsi et al. 2020), whereas others demonstrate clear decline among assessments (Elamin et al. 2013; Beeldman et al. 2020; Bersano et al. 2020). Additionally, it is conceivable that within the cohort of ALS with early cognitive dysfunction, progression may be heterogeneous or restricted to a subgroup, which has been suggested in a small number of longitudinal studies (e.g., Elamin et al. 2013; Mchutchison et al. 2024).

However, this lack of consensus on cognitive impairment progression may also be attributed to various confounding factors within longitudinal studies, including many of those included in a recent meta‐analysis (Finsel et al. 2023). This includes consistently high attrition rates between baseline and follow‐ups, typically between 40% and 60% by 4–9 months (Table 2), which could particularly affect subjects with rapid decline and major cognitive deficits (Consonni et al. 2021). Studies rarely report detailed analyses comparing baseline characteristics of ALS patients who drop out with those who complete follow‐up, typically listing reasons for withdrawal instead. Elamin et al. (2013) found a significant overrepresentation of patients with executive impairment at baseline among those who dropped out, whereas Woolley et al. (2018) observed no link to baseline cognitive dysfunction but did identify an association with more pronounced behavioral issues. Conducting such comparative analyses in all longitudinal trials would help address potential selection bias, improving the robustness of findings.

**TABLE 2:** Summary of longitudinal studies of cognitive dysfunction in amyotrophic lateral sclerosis (ALS) (2013–2023).

Study	Neuropsychological examination(s)	Follow‐up interval	Attrition rate (ALS)^a^	Disease duration at baseline (months)	Results summary	Additional
Elamin et al. (2013)	Standard testing of executive dysfunction, memory, language, and visuoconstruction	3 follow‐ups at 6, 12, and 18 months	Baseline: *n *= 186 Retest 1: 47% Retest 2: 75% Retest 3: 94%	N/A Since diagnosis: 5.0	Decline was fastest in those impaired at baseline. Normal baseline associated with slower motor and cognitive progression	Executive impairment at baseline was most associated with high attrition
Proudfoot et al. (2015)	Revised Addenbrooke's Cognitive Examination	Up to 4 follow‐ups at 4, 6, 12, 18, and 24 months	Baseline: *n *= 61 Retest 1–3: N/A Retest 4: 94%	38.6	No significant progression of cognitive symptoms	
Gillingham et al. (2017)	ALS–CFB	One follow‐up at ∼9 months	Baseline: *n *= 20 Retest 1: 45%	44.4	Executive functioning showed progressive decline	Changes between tests were not associated with disease severity
Bock et al. (2017)	ALS–CBS	One follow‐up at mean 6.8 months	Baseline: *n *= 86 Retest 1: 43%	62.1	No cognitive deterioration	
Burkhardt, Neuwirth, and Weber (2017)	ECAS + FAB	One follow‐up at 12–18 months	Baseline: *n *= 24 Retest 1: 58%	44.4	No cognitive or significant behavioral deterioration	Evidence of practice effects in controls only
Woolley et al. (2018)	ALS–CBS, VFI, COWAT	One follow‐up at mean 11.5 months	Baseline: *n *= 294 Retest 1: 54%	14.3	No cognitive deterioration but progressive behavioral deterioration	
Poletti et al. (2018)	ECAS + FAB, MoCA	Three follow‐ups at 6, 12, and 18 months	Baseline: *n* = 168 Retest 1: 71% Retest 2: 89% Retest 3: 93%	19.0	No cognitive deterioration	Evidence of practice effects in ECAS
Beeldman et al. (2020)	Battery of 13 neuropsychological tests, including ALS‐FTD‐Q for pre‐ and post‐stratification	One follow‐up at 6 months	Baseline: *n *= 35 Retest 1: 20%	8.0	Over a third showed cognitive deterioration (shift to more severe category)	
Trojsi et al. (2020)	ECAS	Two follow‐ups at 6 and 12 months	Baseline: *n *= 22 Retest 1: 23% Retest 2: 36%	15.1	No cognitive deterioration	Despite progressive extramotor functional connectivity decline (fMRI/DTI)
Bersano et al. (2020)	Battery of 12 neuropsychological tests and revised criteria for ALS–FTSD (Strong et al. 2017) for pre‐ and post‐stratification	One follow‐up at 6–8 months	Baseline: *n* = 146 Retest 1: 4%	N/A Since diagnosis: 3.0	Overall, 32% showed worsened cognitive impairment, including 24% unimpaired at baseline. Cognitive decline correlated with ALS–FRS‐R and was associated with shortened survival time	Attrition rate unconfirmed as true baseline *n* unclear
Mchutchison et al. (2024)	ECAS and a semistructured interview to report on behavioral symptoms	Three‐to‐five follow‐ups every 3–6 months	Baseline: *n *= 237 Retest 1: 77% Retest 2: 56%	27.3	On average, no cognitive impairment except in two subgroups characterized by (1) *c9orf72* mutation status and (2) fewer years of education	Disease progression and early closure were the most common reasons for having fewer than three assessments

Abbreviations: ALS, amyotrophic lateral sclerosis; ALS–CBS: ALS cognitive behavioral screen; ALS–CFB: ALS–computerized frontal battery; ALS–FRS‐R: ALS–Functional rating scale revised; ALS‐FTD‐Q; ALS‐FTD‐Questionnaire; bvFTD: behavioral variant FTD; COWAT: Controlled Oral Word Association Test; ECAS, Edinburgh Cognitive and Behavioural ALS Screen; ECAS: Edinburgh cognitive and behavioral screen; FAB: frontal assessment battery; HCs: healthy controls; MoCA: Montreal Cognitive Assessment; PLS: primary lateral sclerosis; PMA: progressive muscular atrophy; VFI: verbal fluency index.

^a^ Attrition rate defined as proportion of ALS participants lost to follow‐up at each retest relative to the original ALS cohort size.

One notable longitudinal study reported significant cognitive decline over a short 4‐ to 6‐month period, with approximately one‐third of ALS patients progressing from cognitively normal to ALSci (Bersano et al. 2020). Although the ECAS was introduced for some participants, a full cognitive battery was also used in this study. Some of the included tests rely on intact physical functioning, and motor symptoms may have progressed during the follow‐up period; however, it is unclear how this was accounted for. Furthermore, it is unknown whether the cut‐offs used to identify impairment adjusted for education, which was, on average, low in this sample. However, this study also stands out for its remarkably early recruitment, with a time since diagnosis of just 3 months at baseline testing—much earlier than other studies. Early recruitment, although logistically challenging, likely provides the best opportunity to detect cognitive changes if present early in the disease process (as discussed in the following section) and may mitigate disease‐associated attrition.

Beyond attrition rates, several other sources of heterogeneity among studies may contribute to the contrasting results observed. These include variations in recruitment strategies (e.g., incidental recruitment of newly diagnosed ALS cases, prevalent or consecutive recruitment), differences in inclusion criteria for patient disease duration, the neuropsychological test batteries employed, follow‐up intervals and frequencies, and the use of control groups (e.g., whether healthy or neurological controls). These variations highlight the clear need for uniformity and standardization of these key variables to enhance trial validity and reproducibility, a point we address toward the conclusion of this review.

Two studies stand out for their methodological robustness, each for different reasons. Elamin et al. was the only study in the table to use a population‐based recruitment strategy, ensuring a broad and representative sample of incident ALS cases (*n* = 186), along with carefully matched controls. Although attrition was relatively high at 47% by the first retest, as previously discussed, a detailed analysis was conducted to compare those who returned for follow‐up with those who dropped out, strengthening the validity of the results. The second mentionable study, McHutchinson et al., had a notably large sample size (*n* = 237) and uniquely accounted for physical symptoms in cognitive assessments. It also used alternate versions of the ECAS to minimize practice effects. Interestingly, both studies found broadly similar results, identifying a subgroup of ALS patients who experienced cognitive decline over time.

Some studies have identified lower cognitive status at baseline (Elamin et al. 2013) or fewer years of formal education (Mchutchison et al. 2024) as an association of subsequent decline. Others identified decline in all groups, including those with initially normal cognition (Beeldman et al. 2020; Bersano et al. 2020). Education level has also been associated with changes in cognition over time such that improvements are seen in those with higher levels (Costello et al. 2021), whereas those with lower levels decline (Bersano et al. 2020; Mchutchison et al. 2024). This relationship between low education and cognitive decline is not specific to ALS and may be interpreted as evidence supporting the theory of cognitive reserve. The concept of cognitive reserve encompasses specific lifelong factors (e.g., socioeconomic status, education, and physical activities) that contribute to an increased number and strength of neural networks, which subsequently makes the brain more resilient to neuropathological damage (Cabeza et al. 2018). An alternative explanation for the role of education is that a higher education level enables the development and utilization of adaptive strategies to maintain performance on cognitive testing (Frankenmolen et al. 2018), thereby masking the presence of cognitive decline.

### 2.4 When Does Cognitive Impairment Begin?

Many of the longitudinal studies of cognition in ALS have shown that cognitive impairment at baseline assessment is common (Finsel et al. 2023). These findings have prompted a growing interest in whether these symptoms begin in the early stages of the disease, prior to motor symptom onset (representing a prodromal disease stage [Benatar et al. 2022]), or whether they may even be neurodevelopmental in etiology (Lulé et al. 2020). To date, much of the research has focused on unaffected C9ORF72 carriers that have highlighted specific areas of poor performance, including letter fluency (Lulé et al. 2020) and cognitive inhibition (Montembeault et al. 2020), with little change in scores over short periods of time. Although these findings highlight a statistically significant difference between unaffected C9ORF72 carriers and non‐carriers, it is unclear whether these differences are clinically meaningful. However, the presence of these deficits has been supported by findings from imaging studies. These include decreased fractional anisotropy that has been shown in the inferior frontal and orbitofrontal brain regions (Lulé et al. 2020), decreased gray matter volume in the cerebellum and insula, and decreased white matter volume in the anterior thalamic radiation (Panman et al. 2019).

Several frameworks aiming to characterize the presence of prodromal cognitive and behavioral symptoms in both ALS and FTD have been proposed (Barker et al. 2022; Benatar et al. 2022; Benussi et al. 2024), but further work involving newly diagnosed or pre‐symptomatic individuals is needed to provide valuable insights into the early development and progression of cognitive symptoms in predisposed individuals. Another potential avenue of exploration is the relatively underutilized group of primary lateral sclerosis (PLS) patients. PLS is a very rare “pure” upper motor neuron form of motor neuron disease (MND). Despite this slower progression, studies have reported similar cognitive and behavioral abnormalities in PLS patients compared to those with ALS (de Vries et al. 2017, 2019). Postmortem analysis suggests PLS is part of the TDP‐43 pathological spectrum, though most consistently focused on the primary motor cortex in the few cases examined (Mackenzie and Briemberg 2020). Therefore, longitudinal cognitive and behavioral studies that assess impairment in appropriately matched patients across the wider MND spectrum that includes PLS might provide valuable mechanistic insights into the development of neuropsychological dysfunction.

### 2.5 Cognitive Dysfunction as a Negative Prognostic Indicator

Several studies have proposed that motor and cognitive manifestations of disease decline in parallel, showing a correlation between cognitive impairment and ALS functional rating scale score (Elamin et al. 2013; Bersano et al. 2020). This was a finding also supported by several cross‐sectional studies (Crockford et al. 2018a; Chiò et al. 2019) suggesting cognitive dysfunction may be a marker of disease severity.

Cognitive dysfunction is also an overall negative prognostic indicator in ALS, associated with more rapid motor function decline and shortened survival (Elamin et al. 2013; Xu et al. 2017). The reasons for this are incompletely understood, although an association between cognitive impairment and bulbar involvement as well as a reduced likelihood for cognitively impaired individuals to opt for life‐prolonging procedures or indeed adhere to any supportive interventions may offer partial explanation (Olney et al. 2005; Caga et al. 2019). Hence, it is imperative that clinicians identify these individuals early to implement supportive measures and empower individuals to make advanced, informed decisions about their future care.

## 3 Behavioral Dysfunction

Numerous screening tools have been devised to assess general behavioral disturbance in ALS, including the ALS‐FTD‐Q questionnaire (Raaphorst et al. 2012) and the Beaumont Behavioural Inventory (Elamin et al. 2017). The Dimensional Apathy Scale has been validated as a reliable and rapid multidimensional assessment of the most prevalent behavioral symptom in ALS (Radakovic et al. 2016). The assessment tool, which can be completed by patients, caregivers, healthcare professionals, or researchers, has been used more recently to investigate clinical correlates of apathy and its subtypes.

Apathy has been associated with cognitive impairment in ALS. Initiation apathy in particular has been strongly correlated with impaired performance in verbal fluency, the most consistent cognitive deficit in ALS (Grossman et al. 2007; Witgert et al. 2010; Radakovic et al. 2017; Kutlubaev et al. 2023). The common etiological basis of this is yet to be fully elucidated, but shared networks, including the dorsolateral prefrontal, inferior, and orbitofrontal cortices, as well as the cingulum, have been postulated as having overlapping functionality across the two faculties (Abrahams 2023). Similarly, one study also found emotional apathy to be significantly associated with emotional recognition on the Ekman 60 Faces talk (Radakovic et al. 2017). As with cognitive dysfunction, an association between behavioral change and bulbar dysfunction is contentious, with several studies concluding in favor (Santangelo et al. 2017; Kutlubaev et al. 2023) and against (Lillo et al. 2011; Consonni et al. 2019). Proposed associations between apathy and depression or other mood disorders are equally as conflicting, although a recent meta‐analysis concluded no clear correlation (Kutlubaev et al. 2023).

There is a general lack of longitudinal studies that comprehensively analyze and track the progression of behavioral symptoms. However, at least two studies conclude behavioral impairment to be progressive either in tandem with (Beeldman et al. 2020) or independently of (Woolley et al. 2018) apparent cognitive deterioration. Although there is evidence to suggest that the severity of behavior symptoms increases, it remains unknown whether the specific types of behavioral symptoms increase or change over time (i.e., does disinhibition decrease as apathy increases).

The presence of behavioral symptoms, including apathy, and even the level of apathy, appears to correlate with worsened ALS prognosis (Hu et al. 2013; Caga et al. 2016; Nguyen et al. 2021; Kutlubaev et al. 2023). Of note and by contrast, one study reported a longer disease duration in those with ALSbi, suggesting that such changes might be more likely to be reported in the later stages of disease (Consonni et al. 2019). There appears to be no consistent correlation between apathy and disease stage (Caga et al. 2016; Kutlubaev et al. 2023).

Likewise, few studies have examined the onset of behavioral symptoms and how these change over time in ALS. Symptoms of disinhibition, perseveration, and apathy have been reported in the early stages of the disease, and in some cases, even before motor symptom onset, based on retrospective reporting (Mioshi et al. 2014). Investigating these symptoms longitudinally in asymptomatic at‐risk individuals is needed to further our understanding of whether specific symptoms occur in the prodromal stages of the disease and if certain behavioral profiles are associated with progression to ALS without significant extramotor involvement, FTD, or ALS–FTD.

## 4 Histopathology

The hallmark pathological feature of 97% of ALS cases is motor neuronal and glial cell cytoplasmic inclusions of a 43 kDa transactive response DNA‐binding protein, TDP‐43. It is predominantly localized to the nucleus in a steady state, but in ALS, it undergoes nuclear clearance, including several aberrant post‐translational modifications that appear to contribute to cytoplasmic mislocalization and aggregation (Suk and Rousseaux 2020). Recapitulating TDP‐43 dysfunction in animal models has become an important focus of research for investigating the motor consequences of TDP‐43 proteinopathy. However, comparatively little research has explored a possible relationship between TDP‐43 and cognitive and behavioral impairment in ALS, primarily due to a lack of validated tools for evaluating TDP‐43 in vivo (Buciuc et al. 2020). The most compelling evidence for a link is that TDP‐43 is also the principal component of inclusions in ∼50% of frontotemporal lobar degeneration (FTLD) cases (FTLD–TDP) and the vast majority of FTLD–ALS cases (Irwin et al. 2015; Kawakami, Arai, and Hasegawa 2019). This suggests a potential continuum from mild cognitive and behavioral impairment in motor‐predominant ALS to frank FTD at the other extreme, in agreement with a large retrospective study that identified an FTLD neuropathological pattern to be the greatest correlate of cognitive impairment in ALS (Borrego‐Écija et al. 2021).

Pathological TDP‐43 deposition has also been associated with cognitive impairment in the absence of FTLD. The recently described limbic‐predominant age‐related TDP‐43 encephalopathy (LATE), which has a more restricted anatomical distribution of TDP‐43 proteinopathy within the temporal lobe, is also associated with substantial amnestic‐like cognitive impairment (Nelson et al. 2019). LATE designation is, however, controversial. Some pathologists cite insufficient evidence for it being a distinct pathology from Alzheimer's disease and FTLD (Josephs et al. 2019). Furthermore, individuals with non‐TDP‐43 neurodegenerative disease exhibiting concomitant TDP‐43 pathology demonstrate increased severity of cognitive impairment compared to those without (Wilson et al. 2013; Meneses et al. 2021). Importantly, the reverse is also true. ALS with concomitant pathologies (e.g., neurofibrillary tangles) appear overrepresented in ALSci cohorts, warranting further investigation into TDP‐43 interdependent pathways as well as potential converging and synergistic pathways of cognitive dysfunction (Strong, Donison, and Volkening 2020; Borrego‐Écija et al. 2021).

Beyond TDP‐43, neurofilament (NF) levels are considered a non‐specific marker of neuroaxonal pathology, and NF levels as measured in cerebrospinal fluid or blood are, on average, higher in ALS cases than in most other neurological conditions (Rosengren et al. 1996; Olsson et al. 2019). NF levels are most consistently linked to the overall rate of neurological disease progression and survival, which is consistent with lower levels being observed in ‘pure’ FTD cases compared to ‘pure’ ALS cases (Lu et al. 2015; Gaiani et al. 2017; Olsson et al. 2019). Given that cognitive impairment is an adverse prognostic factor in ALS, it would be intuitive to expect higher NF levels in such patients. This appears to be the case in several other neurodegenerative diseases, such as Parkinson's disease, and dementias, including FTD (Aamodt et al. 2021; Silva‐Spínola et al. 2022; Lehmann et al. 2023). However, this has not been routinely observed in ALS (e.g., Gaiani et al. 2017; Feneberg et al. 2018), although one study found significantly elevated plasma NF light chain levels in the ALS–FTD cohort compared to ‘pure’ ALS or FTD groups (Vacchiano et al. 2021).

### 4.1 Extramotor Pathology

A neuropathological analysis of 27 non‐demented people with ALS who had been cognitively screened in life using the ECAS identified extramotor TDP‐43 pathology in cognitive domain‐matched brain regions, (e.g., executive dysfunction: orbitofrontal cortex; language dysfunction: inferior frontal gyrus) within all those classified as ALSci (*n* = 7) (Gregory et al. 2020). While this designation may be criticised for being overly regionally reductionist, its significance lies not only in the potential mechanistic underpinning of cognitive dysfunction in ALS by extramotor TDP‐43 proteinopathy, but also in the prospect that the ECAS could be used to pre‐stratify those with extramotor TDP‐43 burden in future clinical trials aimed at reducing TDP‐43 pathology. These findings align with a previous smaller prospective study that also identified a correlation between TDP‐43 pathology in non‐primary motor areas and cognitive dysfunction, especially apparent in ALS–FTD cases (Prudlo et al. 2016). TDP‐43 deposition in behavioral domain‐matched regions (e.g., orbitofrontal cortex, ventral anterior cingulate, and medial prefrontal cortex) was also identified, albeit in the only two ALSbi individuals in the study.

However, extramotor TDP‐43 pathology was also identified in six ALS cases without cognitive or behavioral impairment (ALScn), indicating low diagnostic sensitivity (44%) relative to specificity (100%) for ECAS predicting TDP‐43 pathology (Gregory et al. 2020). This may suggest a more complex relationship between TDP‐43 and cognitive impairment or the possibility of yet‐to‐be discovered ‘resilience’ factors capable of modifying or ameliorating TDP‐43 perturbed pathways. The study's conclusions were further limited by small sample size and variable time differences between ECAS and death. The latter reflects a caveat of all postmortem studies, which reflect end‐stage disease. Nevertheless, the conclusions from this study are an exciting development pending validation in larger disease cohorts. More broadly, the authors provided a robust platform for assessing clinicopathological relationships in well‐phenotyped ALSci or indeed ALSbi cohorts.

### 4.2 Pathological Staging

The identification of patterns of TDP‐43 across postmortem ALS brains has been defined as a series of histopathological “stages” (Braak et al. 2013; Brettschneider et al. 2013). A sequence of development has been inferred beginning with mild pTDP‐43 burden in the motor cortex, motor nuclei, and spinal motor neurons (Stage 1) before spreading to the prefrontal cortex, red nucleus, and striatum (Stages 2 and 3), and later still, the hippocampus (Stage 4). In inferring a “prion‐like” spread of TDP‐43 pathology in a typified, sequential manner, it is crucial to underscore that molecular evidence for an amyloidogenic nature of TDP‐43, plus its ability to self‐template in a prion‐like manner, remains unproven. Histopathological staging, by definition cross‐sectional and postmortem, cannot yet be linked to clinical phenotype with confidence (indeed, the publication explicitly reports no relationship of the staging to any clinical data, most notably including disease duration).

One retrospective study found cognitive impairment in ALS to be most associated with Brettschneider stage 4 (Borrego‐Écija et al. 2021). Puzzlingly though, the same study also identified TDP‐43 pathology in the anterior cingulate cortex (Stages 2 and 3) to be especially associated. An association with generally advanced Brettschneider staging is however consistent with other neuropathological studies emphasizing the importance of TDP‐43 burden in the superior and middle frontal gyrus (Stage 3) (Brettschneider et al. 2012) and hippocampus (Stage 4) (Takeuchi et al. 2016) as possible correlates of cognitive dysfunction in respective ALS cohorts.

It is unsurprising that cognitive involvement does not fit neatly into a histological model of TDP‐43 dissemination based on postmortem data. No clear clinicopathological correlations have been established between pTDP‐43 stage and motor symptom severity (Saberi et al. 2015). Given the pathological overlap between ALS and FTLD, it may be more beneficial to consider both ALS and FTLD staging systems in parallel in future clinicopathological study designs to shed light on the extent to which cognitive dysfunction in ALS can be explained by an FTLD‐like pathological continuum.

Alternatively, TDP‐43 deposits may cause synapse loss in the prefrontal cortex, akin to processes observed in Alzheimer's disease. Intriguingly, synapse losses in cases of cognitive impairment are not necessarily associated with cortical atrophy (Henstridge et al. 2018). Histological projects assessing any relationship among TDP‐43 deposition, synaptic protein levels, and synaptic integrity will be needed to explore this further.

On a mechanistic level, it will be important to elucidate particular pathways of TDP‐43 dysregulation that may underpin cognitive or behavioral dysfunction, be it via neuronal/glial death, synaptic loss, or other maladaptive pathways. As an important RNA‐binding protein, identification of aberrant transcriptomic signatures associated with impaired TDP‐43 proteostasis is especially pertinent. Advances in bioinformatic analysis as well as in spatial transcriptomics will be of benefit here when applied to pre‐stratified cohorts (Mehta et al. 2022). However, it is also conceivable that disease pathways leading to neuropsychological dysfunction may not always have a clear TDP‐43 pathological correlate. Loss of TDP‐43 splicing repression, with the potential translation of cryptic exon‐encoded *neopeptides*, has been hypothesized as an early or even pre‐symptomatic event in ALS/FTD pathogenesis, even in the absence of overt TDP‐43 pathology (Irwin et al. 2024). This highlights the importance of not overlooking brain regions without obvious pathology.

## 5 Genetics

Up to 20% of ALS cases are linked to dominantly inherited pathological variants (Al‐Chalabi, van den Berg, and Veldink 2017). The most common in North American and European populations is an intronic hexanucleotide repeat expansion (HRE) within *C9orf72* (Dejesus‐Hernandez et al. 2011; Renton et al. 2011). This accounts for approximately 40% and 25% of familial cases of ALS and FTLD, respectively (Majounie et al. 2012). *C9orf72*‐related ALS (C9‐ALS) is associated with an earlier age of symptom onset, including both cognitive and behavioral manifestations, in particular significant deficits in language, executive functioning, and behavioral inhibition compared to non‐C9 cases (Byrne et al. 2012; Irwin et al. 2013). Whether this represents a unique endophenotype of C9‐ALS remains uncertain. One study found similar neuropsychological deficits, particularly poorer performance on letter fluency among non‐C9 relatives of familial ALS cases, suggesting that these cognitive traits may reflect a broader endophenotype associated with ALS risk rather than being exclusive to the *C9orf72* repeat expansion (Costello et al. 2023). Another study identified an inverse correlation between HRE size and degree of cognitive impairment (Colombo et al. 2023), but this remains unverified. Comparative structural imaging studies have also confirmed a congruent pattern of extramotor cortical and subcortical (particularly thalamic) involvement consistent with their neuropsychological profile (Bede et al. 2013).

Although a major predictor of cognitive and behavioral dysfunction in ALS, the typical cognitive profile and progression of C9‐ALS may not necessarily reflect the clinical course of sporadic ALSci. A recent, large longitudinal study (*n* = 237) found C9‐ALS individuals were more likely to experience progressive cognitive decline than non‐carriers, for example, (Mchutchison et al. 2024). However, it is worth noting that in this study, not everyone with progressive cognitive decline were C9‐ALS individuals, suggesting that other factors are also responsible for this pattern of change. Some studies have even suggested neuropsychological and psychiatric manifestations (e.g., Snowden et al. 2012; Costello et al. 2023) may precede classical motor symptoms and be an initial presentation of C9‐ALS disease, but this remains unproven (Mioshi et al. 2014; Lulé et al. 2020).

Among other genes associated with both ALS and FTD, *TBK1* is the next most common. Familial ALS, FTD, and ALS–FTD patients with TBK1 pathological variants often exhibit a behavioral phenotype characterized by symptoms, such as behavioral disinhibition, apathy, and emotional lability (Van Mossevelde et al. 2016; Mccombe et al. 2019). Following *TBK1*, pathological variants in *TARDBP* (encoding TDP‐43) and *VCP* are comparatively rare. About one‐third of ALS patients with pathological variants in *TARDBP* present with ALSci/ALSbi or ALScbi, generally lacking distinctive cognitive or behavioral impairment compared to sporadic ALS (Moglia et al. 2024). Conversely, less is clear about *VCP*‐associated ALS, but a review of published cases indicated the predominant phenotype to be ‘classical ALS’ with cognitive or behavioral symptoms reported infrequently (Feng et al. 2022).

The second most common genetic variants associated with North American and European ALS cases are found in *SOD1*, accounting for 20% of familial cases and 2% overall. Such cases notably lack TDP‐43 pathology (Mackenzie et al.^2007^). Although initial studies suggested the absence of cognitive or behavioral impairment in *SOD1*‐related ALS (e.g., Wicks et al. 2009), more recent research has challenged this. When compared to non‐carriers, *SOD1*+ pathological variant carriers have been shown to have significantly poorer performance on measures of executive functioning (Marjanović et al. 2017), social cognition, and language (Chio et al. 2024), as well as higher rates of behavioral impairment (Dalla Bella et al. 2022). Different *SOD1* ALS genotypes may even exhibit distinct cognitive profiles. A recent study reported that although homozygous carriers of the D91A *SOD1* variant showed deficits in some cognitive tasks, particularly those assessing working memory, they significantly outperformed carriers of other *SOD1* mutations and sporadic ALS patients in tasks measuring cognitive processing speed (e.g., Digit Symbol Test) and cognitive flexibility (e.g., Stroop Test), with performance comparable to healthy controls (Winroth et al. 2024).

Behavioral symptoms meeting the criteria for ALSbi were reported in 50% of *SOD1* variant carriers and included more symptoms of mental rigidity and irritability (Dalla Bella et al. 2022). Furthermore, behavioral impairment appeared to be specific to those with variants in Exon 5. Rare cases of ALS linked to variants in *FUS* gene also lack TDP‐43 pathology yet may be associated with FTD (Neumann et al. 2009).

Additionally, future genome‐wide association studies incorporating large, multi‐center cohorts of apparently sporadic ALSci, ALSbi, and ALScn may propose novel gene variants that confer a heightened risk of developing cognitive and behavioral impairment. Indeed, polymorphisms in *UNC13A* (rs12608932) and *TMEM106B* (rs1990622) have both been associated with cognitive impairment in ALS in some (Vass et al. 2011; Tan et al. 2020; Willemse et al. 2023), but not all (Mchutchison et al. 2024) studies, as have *SOD1* variants in Exon 5 (Dalla Bella et al. 2022).

## 6 Neuroimaging

Histopathological findings associated with a particular disease process offer important clues as to the etiological basis of that disease. However, in‐life neuroimaging is crucial to identify neuroanatomical correlates of clinical symptoms with potential biomarker utility. Magnetic resonance imaging (MRI) and positron emission tomography (PET) studies provide evidence for structural, functional, and metabolic tissue changes associated with cognitive dysfunction in ALS. Structural MRI studies in ALS–FTD have detected regional gray matter loss in brain areas with roles in higher neurological functioning, including frontotemporal, cortical, and subcortical (e.g., caudate nucleus) areas, as well as more cortical thinning in more classical regions such as the dorsal motor cortex (Masuda et al. 2016; De Marchi et al. 2021). Within ALSci, cortical thinning is more pronounced in frontal, temporal, cingulate, and insular regions supporting the notion of a cognitive impairment–associated cortical atrophy profile for ALSci individuals, albeit with moderate inter‐individual variation (Schuster et al. 2014; Agosta et al. 2016; Consonni et al. 2019; Consonni et al. 2020). Notably though, extramotor cortical thinning is also present in ALScn cases limiting its specificity (Benbrika et al. 2021). Consistent with the aforementioned evidence for ALSci typically exhibiting more advanced pathological staging, motor cortex atrophy in cognitively impaired individuals is often more severe (Mioshi et al. 2013; Omer et al. 2017).

Neuroanatomically relevant patterns have also been observed for white matter loss as indicated by reduced fractional anisotropy (diffuse‐tensor imaging) in ALSci within tracts important for cognition and behavior, including the corpus callosum, superior longitudinal fasciculus, cingulum, and orbitofrontal gyrus (Evans et al. 2015; Masuda et al. 2016; Cheng et al. 2020). Finally, several fluorodeoxyglucose–PET studies have demonstrated moderate frontal and temporal hypometabolism in FTSD–ALS cases on a continuum with more severe hypometabolism in frank FTLD–ALS cases, in contrast to little or no changes in ALScn (Canosa et al. 2016; Hinault et al. 2022). These results are consistent with a recent functional MRI study, which identified decreased functional connectivity in frontotemporal areas comparing ALSci and ALS–FTD within an ALScn cohort (Temp et al. 2022).

A smaller number of structural and functional brain imaging studies have also been instrumental in identifying key cortical regions and tracts associated with behavioral impairment. Importantly, and as mentioned previously, neuroanatomical correlates of apathy that involve several fronto‐subcortical regions appear to substantially overlap with those also implicated in verbal fluency dysfunction (Woolley et al. 2011; Agosta et al. 2016; Femiano et al. 2018). Similarly, research into the neuroanatomical correlates of disinhibition has identified atrophy in the temporal and cingulate regions of the right hemisphere, as well as the right superior frontal gyrus (Consonni et al. 2019). Compared to our understanding of cognitive dysfunction in ALS, less is known about other behavioral symptoms and their overlap with cognition in ALS highlighting the need for further research in this area.

### 6.1 Longitudinal Studies

Longitudinal MRI studies show conflicting results for microstructural changes in extramotor brain regions associated with ALS disease progression. One study found no additional gray or white matter changes in cognitively impaired individuals (van der Burgh et al. 2020), whereas others found progressive loss and abnormality of gray and white matter volume, which correlated with impaired cognition (Keil et al. 2012; Kwan et al. 2012;Hinault et al. 2022). Intriguingly, another identified significant white and gray matter changes over 9 months but with no accompanying alteration in cognitive functioning (Benbrika et al. 2021). As discussed, many recent longitudinal studies are confounded by selection bias toward cognitively intact individuals able to consent to scanning, and so future efforts should focus on better accommodating ALS–FTSD needs.

## 7 Concluding Remarks

**FIGURE 1:**
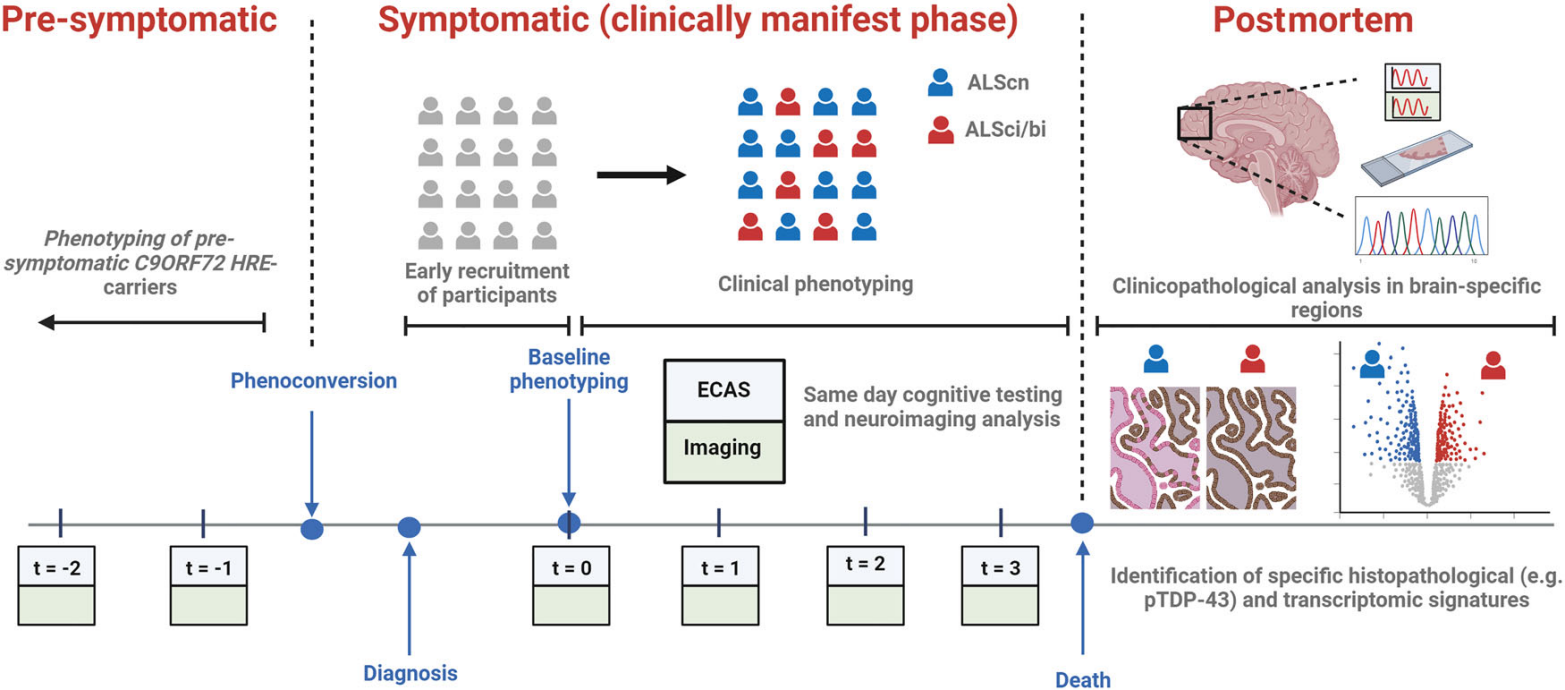
**A potential pipeline for assessing the clinicopathological basis for cognitive and behavioral dysfunction in ALS**. Asymptomatic recruitment of participants is essential. Clinical phenotyping should occur at regular periods from baseline to death using consistent cognitive and behavioral assessment and same‐day neuroimaging analysis to minimize temporal dissociation. Known pre‐symptomatic *C9orf72* hexanucleotide repeat expansion (HRE) carriers can be utilized to elucidate the genesis of cognitive/behavioral impairment in the ALS disease process. At postmortem, pTDP‐43 immunohistochemical staining and transcriptomic analysis (e.g., bulk RNA‐seq and in situ hybridization techniques) on clinico‐anatomically relevant regions may identify pathological signatures associated with cognitive or behavioral domain‐specific impairment. ALS, amyotrophic lateral sclerosis; ECAS, Edinburgh Cognitive and Behavioural ALS Screen. *Source*: Created with Biorender.com.

Although many clinical studies consistently affirm an association between cognitive dysfunction and specific factors such as bulbar involvement, advanced disease and faster rates of disability progression, longitudinal studies have been frequently confounded by selection bias and inappropriate testing platforms with inconsistent conclusions. Robust longitudinal studies focusing on monitoring behavioral dysfunction in ALS are scarce. Clinical correlation and neuroimaging analyses suggest that the most commonly reported cognitive deficit, verbal fluency, is highly associated with the most commonly reported behavioral change, apathy. Cognitive impairment has been linked to the presence of extramotor TDP‐43 deposits in clinico‐anatomically relevant areas. This is substantiated by neuroimaging studies identifying frontotemporal patterns of structural and metabolic deficiencies in cognitively impaired cases. Next steps will need to focus on elucidating important TDP‐43 disease pathways driving these phenotypic consequences. From a genetic perspective, C9‐ALS cohorts represents a leading resource for understanding the pre‐motor cognitive and behavioral landscape through partnership with asymptomatic carrier individuals. A framework for how this might be achieved is outlined (Figure 1).

## Author Contributions

**Alexander Bampton**: conceptualization, writing–original draft. **Caroline McHutchison**: writing–original draft, writing–review and editing. **Kevin Talbot**: writing–review and editing. **Michael Benatar**: writing–review and editing. **Alexander G Thompson**: writing–review and editing. **Martin R Turner**: conceptualization, writing–review and editing, supervision.

## Conflicts of Interest

The authors declare no conflicts of interest.

### Peer Review

The peer review history for this article is available at https://publons.com/publon/10.1002/brb3.70115.

## Data Availability

Data sharing is not applicable to this article as no new data were created or analyzed in this study.
